# Supporting teenagers with period pain in general practice: clinical review

**DOI:** 10.3399/bjgp24X738489

**Published:** 2024-05-31

**Authors:** Sharon Dixon, Neda Taghinejadi, Flora Holloway, Andrew Papanikitas, Katy Vincent

**Affiliations:** Nuffield Department of Primary Care Health Sciences, University of Oxford, Oxford.; Oxfordshire Sexual Health Service OUH, Nuffield Department of Women’s Reproductive Health, Oxford.; School of Social and Political Sciences, University of York, York.; *BJGP*; Honorary Tutor in General Practice, Nuffield Department of Primary Care Health Sciences, University of Oxford, Oxford.; Nuffield Department of Women’s and Reproductive Healthcare, University of Oxford, Oxford.

## Introduction

Period pain (dysmenorrhoea) affects approximately 70–90% of teenagers who menstruate; one-third report marked pain and 20–30% regularly miss school. When at school, menstrual pain impacts on schoolwork and contributes to missing social and sporting opportunities.^1^^–^^3^ Despite evidence-based treatment options, worldwide literature suggests that most adolescents do not seek health care.^4^ Reasons include thinking period pain is normal, which can be reinforced if family and friends experience similar pain, or distrust or wariness of healthcare professionals (HCPs), and not knowing whether their pain is serious enough (or will be taken seriously).^5^ Although adolescents are encouraged to seek advice if their pain is abnormal,^6^ this can be a difficult assessment for teenagers to make. A recent survey of 442 UK school students found that over a quarter did not know whether their periods were normal or not, and that 29.5% had seen an HCP about their periods.^7^ Health professionals also experience uncertainty about when menstrual pain is normal, or not.^8^^,^^9^ Talking about menstruation can be experienced as difficult or stigmatised, and occurs within ‘menstrual etiquette’ governing how and when menstruation is discussed.^10^

## Dysmenorrhoea: symptoms and natural history

Other symptoms can be associated with dysmenorrhoea, including fatigue, altered bowels or appetite, bloating, dizziness, headaches, emotional changes, and sleep disruption. These most commonly occur with the onset of menstrual bleeding, but can precede this by a few days. Menstrual pain can affect both ovulatory and non-ovulatory or regular and irregular cycles.^5^

The natural history of adolescent dysmenorrhoea is uncertain, but available evidence suggests it tends to increase and peak during adolescence with both chronological and gynaecological age, then fall into adulthood.^5^ This statistical trend may not relate to individual experiences.

## Potential underlying or contributory causes for adolescent dysmenorrhoea

Menstrual pain can occur in the absence of identified pelvic pathology (primary dysmenorrhoea) or in association with underlying conditions (secondary dysmenorrhoea).

Clinical pathways are often centred on navigating a clinical distinction between these,^11^ but, in reality, this is not always straightforward or evident, making careful counselling and follow-up for any intervention vital. It is also important to not risk creating a perceived hierarchy of validity for care. Menstrual pain is associated with central nervous system sensitisation,^12^ and the widespread impacts on education, quality of life, and wellbeing make it important to create spaces for all young people experiencing impactful dysmenorrhoea to have opportunities for care.

However, it *is* important to consider possible associated or contributory conditions. In adolescence, the most common cause of secondary dysmenorrhoea is endometriosis, and adenomyosis may be more common than previously thought.

Endometriosis in adolescence is increasingly recognised, and dysmenorrhoea is an important presenting symptom. The community prevalence of adolescent endometriosis is unknown, with evidence predominantly from specialist surgical centres.^1^ However, many people diagnosed in adulthood with endometriosis recall symptom onset in adolescence.^1^ Adolescents may experience different symptoms from adults, for example, more non-cyclical pain and nausea, and are less likely to present with sub-fertility. Both endometriosis and dysmenorrhoea are associated with a positive family history respectively. Given the likely incomplete ascertainment of endometriosis diagnoses, it is suggested that teenagers with dysmenorrhoea should be asked about a family history of both endometriosis and marked dysmenorrhoea. Adolescents with endometriosis describe complex journeys through diagnosis and care.^5^

Other important considerations in adolescence include pelvic inflammatory disease, ovarian cysts, and congenital urogenital tract anomalies.^5^ Congenital anomalies of the urogenital tract can present with dysmenorrhoea in adolescence. The pain is often severe with onset from menarche, can be unilateral, and can be associated with other urogenital developmental anomalies. Congenital anomalies are associated with a higher likelihood of endometriosis^5^ and may require surgical management (for example, treatment of a blind-ending uterine horn) to improve symptoms.

## How should a young person with menstrual pain be assessed in primary care?

**Figure 1.**
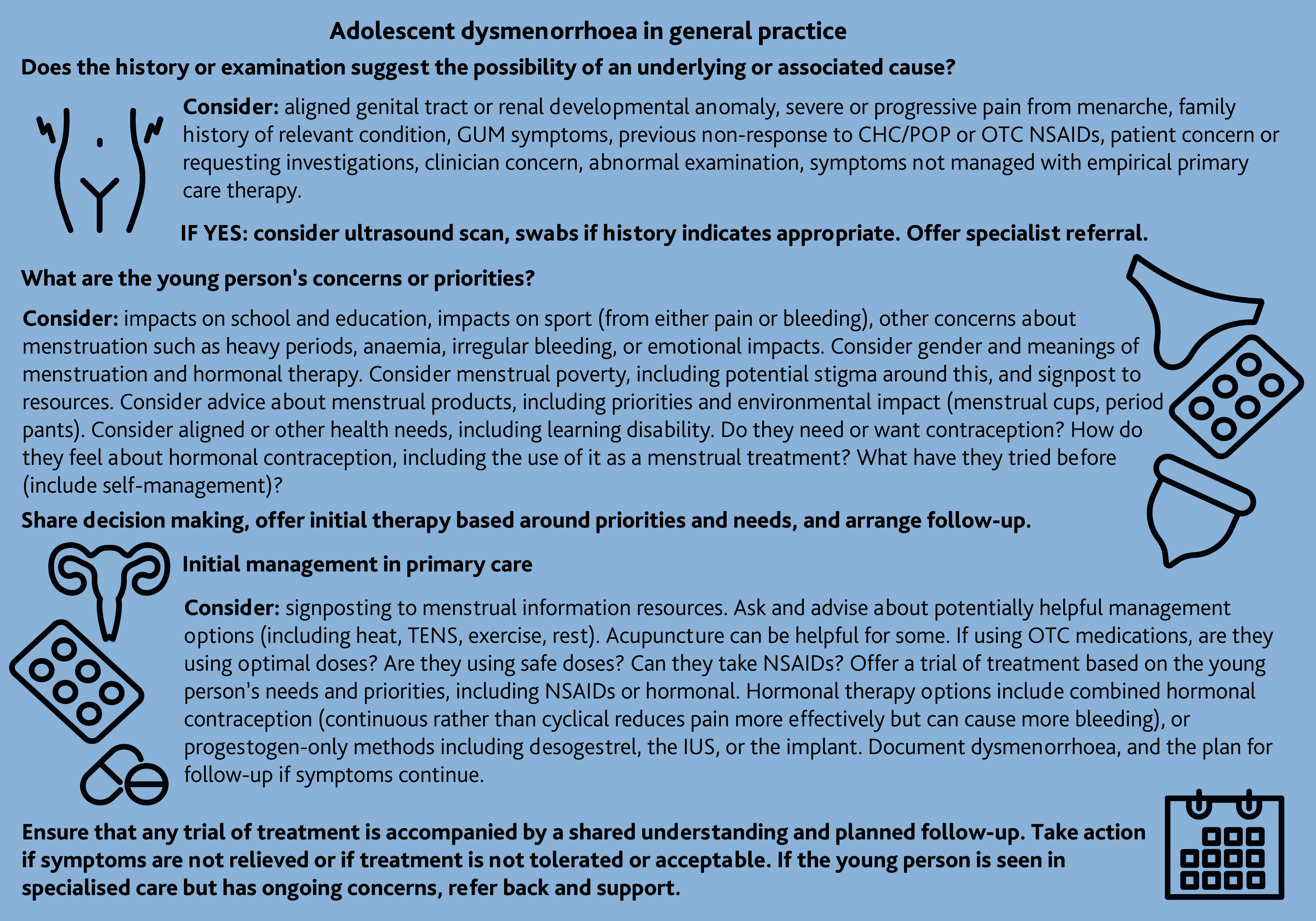
Summary of potential considerations when supporting teenagers with dysmenorrhoea in general practice. CHC = combined hormonal contraception. GUM = genitourinary medicine. IUS = intrauterine system. NSAIDs = non-steroidal anti-inflammatory drugs. OTC = over the counter. POP = progestogen-only pill. TENS = transcutaneous electrical nerve stimulation.

Potential considerations when exploring adolescent dysmenorrhoea in general practice are summarised in Figure 1. While being aware that periods can be difficult to talk about, asking young people about how period pain affects school attendance and engagement, or their hobbies and social activities, and validating their experiences and concerns, can highlight impactful symptoms.^5^

Menstrual pain and pre-menstrual syndrome/pre-menstrual dysphoric disorder can coexist and exacerbate each other, which is worth exploring. Menstrual pain may be exacerbated by smoking.^5^

Menstrual pain can be associated with heavy menstrual bleeding, which may complicate menstruation management.^13^ Exploring the relative contributions of pain and bleeding when considering management options is important. Products such as menstrual cups and underwear can help manage menstrual bleeding with less environmental impacts. Being aware of possible impacts of period poverty and potential stigma around this is important, but asking creates opportunities for support. There is a national programme to mitigate against menstrual poverty.^14^

Menstrual pain and bleeding are important considerations when supporting families and patients living with physical and learning disabilities;^5^ asking about cyclical behavioural or mood disturbance during learning disability checks creates opportunities for supportive intervention.

While an area where more evidence is needed, it is important to ask about experience and impacts of menstrual pain in trans-masculine adolescents.^15^ This may warrant specialist advice if they are taking hormonal medications.

It is the experience of the current authors that apprehension about intimate examination represents a significant barrier to seeking health care. Examination should be undertaken if it is likely that this will significantly change clinical management, for example, with possible pelvic inflammatory disease. If not likely to change management, the GP should consider carefully if this is needed or necessary. If so, the young person should be given autonomy to choose who, where, and when this will occur. While guidance on identifying adolescent endometriosis advises rectal examination, if vaginal examination is deemed unacceptable, the current authors question the sensitivity and acceptability of this in general practice, and do not advise it.

If there is any concern about possible associated causes, for example, because of personal or family history, concerning or marked symptoms, or non-response to a trial of treatment, then the young person should be referred for a pelvic ultrasound scan and specialist assessment, aligned with available services. An ultrasound scan might show a congenital anomaly or endometrioma, but it is important to remember that a negative scan does not rule out endometriosis, and referral should still be arranged if there are concerns.

## What treatment should GPs offer?

The priorities and needs of the young person influence which treatment options are offered or considered acceptable. This includes preferences around hormonal treatment, and whether they need or want contraception. If hormonal contraception is not an acceptable option, and alternatives are not effective or possible, guidance suggests considering specialist referral.^16^

Any intervention trialled for adolescent dysmenorrhoea should be followed up, and young people should have a clear understanding of their options if treatment is ineffective. Young people with debilitating or difficult symptoms after a trial of treatment, *or* for whom a trial of treatment is contraindicated, unacceptable, or not tolerated, should be offered specialist referral.^16^

Self-care and non-pharmacological strategies can be helpful. A Cochrane review demonstrated that exercise is an effective intervention for dysmenorrhoea,^17^ although accounts from young people illustrate that rest and exercise avoidance are also well-documented self-care strategies. External heat application and TENS machines can be beneficial. There is conflicting evidence for acupuncture, but some may find this helpful. There is no consistent evidence for dietary supplements, but replacing vitamin D if deficient can be valuable.^5^

Non-steroidal anti-inflammatories are an evidence-based treatment for dysmenorrhoea.^5^ While embedded in guidance, and effective for some, there is less trial evidence for their effectiveness in endometriosis-associated pain, reinforcing the importance of ensuring follow-up after trials of treatment.

Hormonal treatments are effective in reducing dysmenorrhoea. Combined hormonal (CH) treatment is the best studied. Continual rather than cyclical CH treatment has a therapeutic advantage though can be associated with irregular bleeding.^5^ Progestogen-only methods that reduce ovulation (for example, desogestrel, implant, or intrauterine system [IUS]) are often used, and clinical experience testifies to their effectiveness, although there is little trial evidence in adolescents.

## Conclusion

Menstrual pain is common and impactful on activities that are critical to teenagers’ wellbeing and social/educational development. Promptly treating and validating all menstrual pain can make a major difference to a teenager now and in their future life. GPs need to keep their doors and minds open to ensure that, if initial therapeutic approaches are not effective, tolerated, or acceptable, opportunities can be created to review the young person and consider next steps.
